# Energy-Efficient Privacy Protection for Smart Home Environments Using Behavioral Semantics

**DOI:** 10.3390/s140916235

**Published:** 2014-09-02

**Authors:** Homin Park, Can Basaran, Taejoon Park, Sang Hyuk Son

**Affiliations:** 1 Department of Information and Communication Engineering, Daegu Gyeongbuk Institute of Science and Technology (DGIST), 333 Techno Jungang-Daero, Hyeonpung-Myeon, Dalseong-Gun, Daegu 711-873, Korea; E-Mails: andrewpark@dgist.ac.kr (H.P.); son@dgist.ac.kr (S.H.S.); 2 Northern Cyprus Campus, Middle East Technical University, Mersin 10, Turkey; E-Mail: basaran@metu.edu.tr

**Keywords:** privacy, wireless sensor networks, activities of daily living

## Abstract

Research on smart environments saturated with ubiquitous computing devices is rapidly advancing while raising serious privacy issues. According to recent studies, privacy concerns significantly hinder widespread adoption of smart home technologies. Previous work has shown that it is possible to infer the activities of daily living within environments equipped with wireless sensors by monitoring radio fingerprints and traffic patterns. Since data encryption cannot prevent privacy invasions exploiting transmission pattern analysis and statistical inference, various methods based on fake data generation for concealing traffic patterns have been studied. In this paper, we describe an energy-efficient, light-weight, low-latency algorithm for creating dummy activities that are semantically similar to the observed phenomena. By using these cloaking activities, the amount of fake data transmissions can be flexibly controlled to support a trade-off between energy efficiency and privacy protection. According to the experiments using real data collected from a smart home environment, our proposed method can extend the lifetime of the network by more than 2× compared to the previous methods in the literature. Furthermore, the activity cloaking method supports low latency transmission of real data while also significantly reducing the accuracy of the wireless snooping attacks.

## 1. Introduction

Smart home research and development, although initiated in the late 90s, has been advancing rapidly over the last few years as the sensors get smarter, smaller and cheaper. It is now possible to monitor most of the phenomena associated with the activities of daily living (ADL) with a small cost. As such, various smart home systems can be found in the domains of healthcare, activity automation, energy conservation and remote access services [1,2]. Parallel to these advancements, problems regarding privacy for residents are also being raised and addressed. Wireless sensors deployed in residential environments introduce vulnerabilities that can leak private information about the residents; hence, privacy protection becomes critical. According to recent studies, one of the major obstacles to the adoption of smart home systems is privacy concerns [3]. This work focuses on protecting confidential information in the smart home environments where battery-powered wireless sensors are deployed to monitor and analyze the behavioral patterns of the residents for various smart services.

The activities within a smart home environment can be identified by analyzing the transmission behavior of the wireless sensors, e.g., using WiFi, Bluetooth (low energy) and ZigBee technologies, without even looking into the information being transmitted [4]. This method can be used by an attacker (with highly sensitive eavesdropping devices) to breach the privacy of individuals living in environments that are deployed with wireless sensors. The state-of-the-art protection mechanisms against such attacks mainly hide the data traffic patterns by sending sensor data at uniform transmission slots. This creates the problem of having to generate fake readings in order to ensure uniformity when no sensor data is available at a scheduled transmission slot. Additionally, real data packets that arrive between slots have to be delayed for the same reason. Although it is possible to protect against attacks based on traffic analysis by concealing the actual transmission patterns, protection schemes decrease the usability of the smart home technologies. For example, fake data transmissions incur considerable energy overhead and increase the maintenance costs for battery-powered devices, while arbitrary data injection delays increase the response time of the system, and this may neither be acceptable in mission critical healthcare systems nor desirable due to significant negative impact on the user experience. Therefore, it is necessary to improve the overhead of privacy protection mechanisms in order to increase the value of smart services. We claim that latency and energy efficiency are two important performance metrics that can be improved while not increasing the vulnerability of the privacy of smart home inhabitants.

We propose an energy-efficient, light-weight, low-latency privacy protection scheme based on generating a small amount of fake data to protect the activity information. Inspired by [5,6], which use the concept of semantic similarity, a selected group of sensors generate fake data that are semantically similar to the real phenomena that is being observed. These dummy activities together form an activity cloak that hides the actual private activities. Methods based on fake data injection make frequent transmissions without considering the semantics of the information visible to the adversary [7,8]. However, the distribution of activities throughout the day are generally similar in home environments, and this fact can be exploited by the adversary; hence, the attack does not start with zero information, and fake data flooding may not reduce the information leakage below this statistical level. For example, in the early hours of the day, the adversary expects activities in the bathroom, kitchen and the bedroom. With some additional knowledge, such as the job of the residents, the adversary can further reduce the number of possibilities. Generating fake data from all of the sensors may result in excessive dissipation of precious energy without any additional benefit. The proposed method tries to hide the actual phenomena by cloaking it with other activities that have high expectancy of occurrence, considering the habits of the inhabitants of a particular environment, within the time window of measurement. This approach significantly reduces the energy overhead of privacy protection. In our experiments, we observe up to a 50% reduction in the energy consumption overhead compared to earlier studies, while keeping the level of ADL leakages to a minimum.

The approach described in this paper exploits the difference between probabilistic distributions of activities over a 24-h period. These distributions depend on the habits and behavioral patterns of the individuals. Since changes in the measurements are consequences of one or more activities, we assume, and experimentally show that data transmission patterns follow activity distributions, which is, in fact, the basis of a side channel attack, which refers to an indirect or statistical/analytic attack to exploit leaked information, *i.e.*, activity distributions, rather than directly using the main source of communication [4]. For example, the probability of a contact sensor deployed on the refrigerator door transmitting data is higher just before or during lunch or dinner time, compared to midnight. We use the earth mover's distance (EMD) metric [9,10] to determine the difference between probabilistic distributions of different sensors and interpret this distance as the semantic difference between sensors, sensor clusters and activities. That is, two distributions with a small EMD are semantically similar. During an activity, the proposed approach generates fake data from semantically similar sensor clusters, so as to limit the information leakage to the attacker by what is already known before the attack. We evaluate this method in terms of the amount of the information leakage, delay overhead and energy consumption of the system, as well as the level of privacy protection, which is measured by the accuracy of attacks. According to our experiments on data collected from smart home testbeds, our proposed privacy protection method increases the network lifetime by more than two-folds, while also providing better privacy protection. Moreover, we observe up to an order of magnitude reduction in real-data latency compared to the state-of-the-art methods.

The main contributions of this work is as follows.

- We propose a novel privacy protection mechanism based on mimicking fake activities that have similar characteristics to the observed set of activities.
- The proposed method exposes the trade-off between energy dissipation and privacy protection via the system parameter *θ*. Various values for *θ* can be used depending on the necessity of the environment or inhabitants to bias towards privacy or energy efficiency.
- The proposed method provides activity-level protection with very little overhead by exploiting the nature of data traffic obfuscation techniques to abstract out an activity as a set of sensors. This abstraction allows us to reflect fake activities without applying any activity recognition algorithms.

The rest of this paper is organized as follows. In Section 2, we explain the process of activity detection via snooping wireless data transmissions. Section 3 illustrates the proposed semantic privacy preservation method in detail. Section 4 presents the experimental settings and evaluation results. In Section 5, we summarize the related work, and in Section 6, we discuss our conclusions and possible future directions.

## 2. A Side Channel Attack for ADL Detection

A side channel attack is an indirect or statistical/analytic attack that exploits information leaked by means of supporting the main source of communication rather than depending on it directly. We assume that the sensor data is protected by a sufficiently secure and lightweight encryption [11,12], so the adversary collects radio fingerprints and timing information in order to infer events and activities within the environment of interest. The attack we describe here is the fingerprint and timing-based snooping (FATS) attack [4], which is a multi-tier scheme consisting of four tiers:

Tier 0 detects unique data sources based on their radio fingerprints. This tier depends on the differences in the radio signatures of different radio transmitters to single out each unique sensor [13].
Tier 1 clusters sensor nodes by analyzing time intervals between radio transmissions. This tier assumes that sensors that are spatially or logically close together fire at close proximity in the time domain. These clusters form logical groups by their locations, e.g., a room, or by their purpose. For example, a group containing the washing machine and the detergent box is formed by purpose, *i.e.*, laundry, and this cluster will be formed even if the washing machine is actually located in the kitchen. The adversary can detect the presence of the individuals by analyzing outputs from Tiers 0 and 1.
Tier 2 analyses the features of the extracted clusters in order to label each cluster. These labels, such as kitchen and bathroom, refer to a logical categorization rather than referring to a strictly physical location, such that the washing machine from the previous example will go into the laundry cluster. After Tier 2, the attacker can predict the location of the residents within the environment with high accuracy [4].
Tier 3 labels each individual sensor based on the feature vectors of sensors extracted from the training data and the cluster labels from Tier 2. For example, the adversary expects to find a refrigerator sensor in the kitchen cluster and compares the features of the sensors classified to be in the kitchen with the features of the refrigerator sensor from the training data. After successfully labeling of clusters and sensors, most of the activities can be detected in real-time [4].

**Figure 1.**
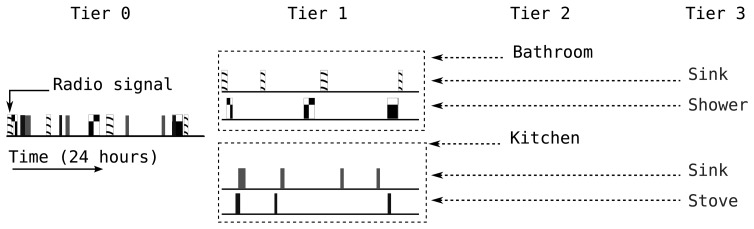
Four tiers of the fingerprint and timing-based snooping (FATS) attack.

Figure 1 summarizes the process followed by the FATS attack. In our FATS implementation, we used one week training data collected from the Aruba smart home testbed [14]. We assume that the Tier 0 attack has been successful, and each unique data source has already been identified. We support this assumption with the previous work on radio fingerprinting [15–17]. For Tier 1, we form sensor clusters based on the temporal distance between the transmission of different sensors. The average time intervals between any pair of sensor transmissions form elements in the Euclidean distance matrix. By applying multi-dimensional scaling (MDS) [18] on the distance matrix, we project the time domain information onto an imaginary two-dimensional plane and obtain *x*, *y* coordinates for the sensors on this plane. Application of k-means clustering on these coordinates forms logical clusters [4,19]. For Tier 2, we first extract feature vectors of the cluster formations. As features, we use:

- median inter-transmission delay (ITD) in the cluster;
- median ITD per sensor in the cluster;
- the number of cluster activities over different periods;
- mean time interval between cluster activities;
- the mean time distance to the next activity of another cluster.

We compute a min-cost bipartite matching between the feature vectors extracted from the Tier 1 clusters and the training data set where the cost of a matching is defined by the Euclidean distance between feature vectors. For Tier 3, sensor pairs from the Tier 2 clusters and the training data are matched based on their features via a linear discriminant analysis (LDA) classifier [20]. The classification process only considers sensors forming the clusters with the same label from the two data sets. Through this process, we annotate each sensor in each cluster with labels, such as a kitchen-doorway sensor, or a bathroom-sink sensor.

In order to recognize activities, we individually process each labeled cluster and apply the LDA classifier on feature vectors summarizing them. These features include information specific to certain sensors, thus making use of the sensor labels generated for the observed environment. As activity feature vectors, we use the mean inter-transmission time, transmission start and end times and activity duration. These features are extracted from both clusters and sensors in clusters.

The method we propose to protect against the aforementioned FATS attack mainly targets the last tier. We claim that activity level protection is both cost effective and safer compared to possible methods working at lower levels. It is safer, because if lower-level information is obtained by other means, e.g., physical inspection of a facility, activity information can be extracted with relative ease. In contrast, activity-level protection does not depend on the physical structure being unknown. The cost efficiency of activity-level protection will be discussed in detail in Section 4.

## 3. Semantic Privacy Preservation via Energy-Efficient Traffic Generation

**Figure 2.**
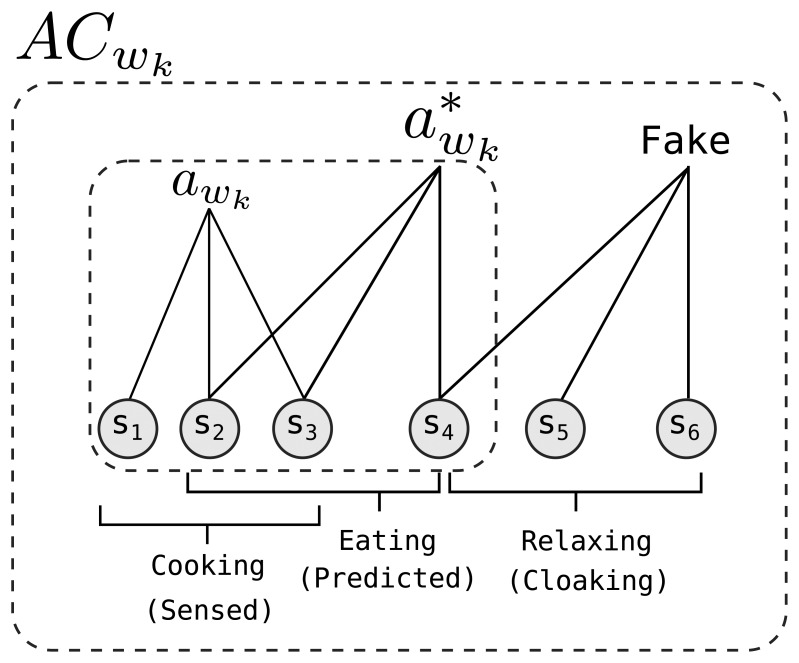
Formation of *AC_w_k__*.

**Figure 3.**
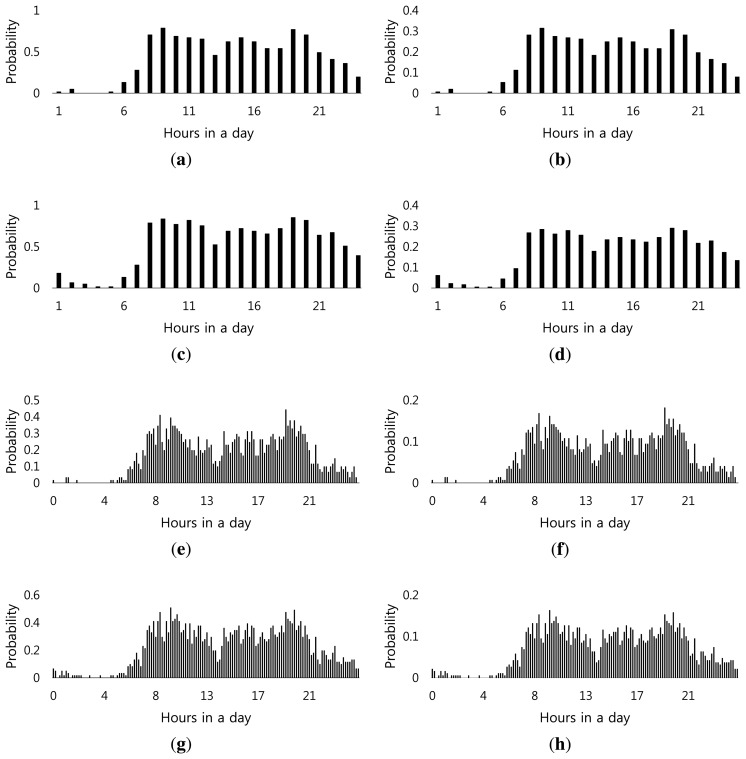
Probability mass functions (PMF) for kitchen and dining room sensors. (**a**) PMF for the kitchen sensor with *w_k_* = 1 h; (**b**) normalized PMF for the kitchen sensor with *w_k_* = 1 h; (**c**) PMF for the dining sensor with *w_k_* = 1 h; (**d**) normalized PMF for the dining sensor with *w_k_* = 1 h; (e) PMF for the kitchen sensor with *w_k_* = 10 min; (**f**) normalized PMF for the kitchen sensor with *w_k_* = 10 min; (**g**) PMF for the dining sensor with *w_k_* = 10 min; (**h**) normalized PMF for the dining sensor with *w_k_* = 10 min.

We define an activity cloak (AC) as a set of activities, including predicted, sensed and fake activities. Since lots of activities happen regularly, each incurring bursty radio transmissions, we are able to pre-compute a set of activities that will soon take place, by both: (1) predicting them from the past history of residents' behavior (predicted activities); and (2) considering subsequent transmissions of current sensor firing (sensed activities). An AC hides these activities in the smart home from the eavesdroppers by tainting them with fake, *i.e.*, cloaking, activities. Since sensor-based activity identification depends on the behavior of a group of sensors, we frequently use the term AC to refer to a set of sensors. Our proposed scheme periodically recalculates and updates the active AC. The interval between two updates is called a time window, *w*. For the *k^th^* time window *w_k_*, the system uses *AC_w_k__*. The length of a time window is a system parameter, and it is fixed during run-time. Accordingly, the number of time windows in a day is fixed, and *w_k_*_+1_ = *w*_0_ for the last time window of the day. Within *w_k_*, only the sensors included in the *AC_w_k__* are allowed to transmit sensor data. By enforcing this transmission behavior, our scheme aims to increase privacy in an energy-efficient manner. We denote the sensed activity in the environment during *w_k_* by *a_w_k__* and the set of predicted activities by $a_{w_{k}}^{*}(a_{w_{k}}\subset a_{w_{k}}^{*}\subset AC_{w_{k}})$, as shown in Figure 2. We introduce and define the term *a_w_k__* to clarify the subsequent definitions, when, in fact, *a_w_k__* is a part of the prediction process. The generation of the activity cloak is completed in three steps:

Step 1 Activity prediction based on the long term history: the long-term resident behavior is analyzed to create a list of sensors that are expected to detect and report an event during *w_k_*, thus to be added to the prediction set $a_{w_{k}}^{*}$. We use a threshold to infer sensors that will likely sense an event during *w_k_*, *i.e.*, each sensor with a probability higher than the threshold is added to $a_{w_{k}}^{*}$. We set this threshold to a fixed value beforehand. The prediction accuracy of $a_{w_{k}}^{*}$ highly depends on a control parameter, *w_k_*, since introducing more time resolution by decreasing *w_k_* lowers the probability of each time window, as shown in Figure 3a and Figure 3e. This performance degradation is due mainly to the fact that predicting actual sensor utilization during a shorter period (e.g., 10 min) is relatively harder than that during longer periods (e.g., 1 h). Comprehensive performance evaluation results to support this observation will be presented in Section 4. To illustrate the likelihood of sensor emission for each time window, Figure 3a and Figure 3e show the probability of motion sensors in the kitchen and the dining room, respectively, firing throughout the day based on the data collected from the CASAS (Center for Advanced Studies in Adaptive Systems) project Aruba smart home testbed [14]. The similarity between two different sensors indicates their relatedness. Note that our system does not try to identify activities online; it simply assumes that sensors that are added to $a_{w_{k}}^{*}$ indicate either a single activity or multiple activities. We assume that an attacker is expecting to see these activities before the attack. For example, in the morning hours, the adversary may expect to see events in the kitchen or in the bathroom.
Step 2 Expanding the prediction set with the sensed activities: The $a_{w_{k}}^{*}$ set from Step 1 is not guaranteed to contain all sensed activities, because there exist unusual activities; in the early hours of the day, the residents may unexpectedly be watching television. We thus use the *a_w_k__* set to accumulate sensors that are actively detecting such events. In order to create the *a_w_k__* set, the central controller (CC) polls sensors at the end of each time window. If a sensor *s_i_* has detected an event during *w_k_*, it requests to be included in the new cloak, *AC_w_k__*__+1__; otherwise, a negative response is sent to the CC. Upon receiving a request message from *s_i_* in the polling period, CC adds *s_i_* to the *a_w_k__*__+1__. The polling period enforces a uniform transmission pattern, so that an attacker eavesdropping on wireless transmissions does not see any irregularities as a result of ongoing activities. At the end of the polling period, *a_w_k__*__+1__ is added to $a_{w_{k+1}}^{*}$. Note that Step 2 ensures that all of the residents' activities are protected by our scheme at the expense of additional energy; it is important to make better activity prediction to further save energy.
Step 3 Addition of the cloaking activities: following Step 2, the $a_{w_{k}}^{*}$ set contains all sensors predicted to detect an event for *w_k_*_+1_. Sensors in $a_{w_{k}}^{*}$ are then placed in *AC_w_k__*__+1__, so that the activity cloak includes predicted activities (Figure 2). Next, *AC_w_k__*__+1__ is expanded with fake activities that are semantically similar to those indicated by the items in *AC_w_k__*__+1__. Instead of adding individual sensors to the cloak, the system adds them in clusters. For this purpose, a list of identified activities is kept, where each activity is defined by a set of sensors. To create the list of activities, we cluster long-term sensor histories using a naive Bayesian classifier. However, classifiers based on the hidden Markov model, conditional random fields or similar classifiers can be used for the same purpose [21,22]. Adding sensors in clusters results in a semantically-sound cloak. For example, the motion sensor in the kitchen is coupled with the one in the dining room, so that they form a fake activity. The process of selecting semantically similar activities is explained in Section 3.1.
  After generating the *AC_w_k__*__+1__, CC broadcasts this information to the sensor nodes. During *w_k_*_+1_, *s_i_* ∈ *AC_w_k__*__+1__ transmits data according to a Fit Probe Rate (FPR) schedule [23], such that an eavesdropper sees a uniform transmission pattern according to some exponential distribution that is independent of the activities. According to FPR, each transmission has to follow this exponential distribution while low latency transmissions are supported by scheduling actual data packets with the minimum possible delay that fits into the distribution. During *w_k_*, the sensors in *AC_w_k__* follow the steps given in Algorithm 1, while all other sensors only record any detected events to prepare themselves for the next AC, *AC_w_k__*__+2__.

**Algorithm 1:** The algorithm executed by *s_i_* ∈ *AC_w_k__*.
 **at**
*each FPR slot*
**do**  send fake data; **end** **at**
*each sensing event*
**do**   Δ*t* = getMinRequiredDelay();  sleep for Δ*t*;  send real data; **end**
  Since our method only allows sensors in the *AC_w_k__* to transmit data during the *w_k_*, a reading from a sensor that is not included in the *AC_w_k__* is delayed until *w_k_*_+1_. Any such sensor reading is called an unexpected event. Since our method handles sensed activity requests while forming the cloak, the upper bound for the delay suffered by an unexpected event will be the time window size. We expect these unexpected transmission requests to be rare because of the long-term predictions and, also, because we keep active sensors in the cloak as long as they are active.

### 3.1. Semantic Distance between Sensors

CC determines the fake activities in *AC_w_k__* based on their semantic similarity to $a_{w_{k}}^{*}$. We use the EMD metric to measure the semantic similarity between activities. Since activity detection is based on clustering readings from different sensors, the semantic distance between two activities is a function of the difference of transmission behaviors of sensors in these activities. Let us denote the probability function of *s_i_* by *P_i_*(*w_k_*) = *p_ik_*, such that *p_ik_* is the probability of *s_i_* sensing an event in *w_k_*. The plot of *P_kitchen_*(*w_k_*) is given in Figure 3a and Figure 3e. EMD requires its inputs to be normalized histograms in order for the relation to be reflexive. Therefore, we define *P̂_i_*(*w_k_*) *= P̂_ik_* as the normalized *P_i_*(*w_k_*), such that:

(1) $${\hat{p}}_{ik}=\frac{p_{ik}}{\underset{k\in n}{\text{∑}}p_{ik}}$$

where *n* is equal to the number of time windows in a day. Given two input probability mass functions (PMFs), e.g., Figure 3b and Figure 3f, assume that one of them is a collection of piles of mud, while the other is a collection of holes in the same space. EMD measures the minimum work required to fill the holes with earth, where the work is defined as transporting a unit of earth over a unit of ground distance. Each such move from point *k*_1_ of the mud piles to the point *k*_2_ of the holes is called a flow, *F*(*k*_1_, *k*_2_) = *f_k_*__1__*_k_*__2__, of mud. We use the magnitude of the difference between points *k*_1_ and *k*_2_ as the distance, *i.e.*, *D*(*k*_1_, *k*_2_) = *d_k_*__1__*_k_*__2__ = |*k*_1_ − *k*_2_|. Given a pair of PMFs, *P̂*_1_ and *P̂*, and a set of flows, *F*, the work due to *F* is computed as follows:

(2) $$W({\hat{P}}_{1},{\hat{P}}_{2},F)=\sum_{k_{1},k_{2}\in n}f_{k_{1}k_{2}}\times d_{k_{1}k_{2}}$$

subject to constraints:

(3) $$f_{k_{1}k_{2}}>0$$

(4) $$f_{k_{1}k_{2}}\leq {\hat{p}}_{1k_{1}}$$

(5) $$f_{k_{1}k_{2}}\leq {\hat{p}}_{2k_{2}}$$

(6) $$\sum_{k_{1},k_{2}\in n}f_{k_{1}k_{2}}=min(\sum_{k_{1}\in n}{\hat{p}}_{1k_{1}},\sum_{k_{2}\in n}{\hat{p}}_{2k_{2}})$$

The first constraint ensures that a flow transfers a positive amount of earth. The second and the third constraints limit the transfer size by the available mass of earth in a given pile and the capacity of the hole at the destination. The last constraint specifies that either the total mass is moved or all of the holes are filled. Based on Equation (2), EMD finds the set of flows, F*(*k*_1_, *k*_2_), that fills the holes with the minimum amount of work and returns the distance between inputs:

(7) $$\text{EMD}({\hat{P}}_{1},{\hat{P}}_{2})=\frac{\underset{k_{1},k_{2}\in n}{\text{∑}}f_{k_{1}k_{2}}^{*}\times d_{k_{1}k_{2}}}{\underset{k_{1},k_{2}\in n}{\text{∑}}f_{k_{1}k_{2}}^{*}}$$

The semantic distance between two sensors can be used to define the semantic distance between sets of sensors, *i.e.*, activities. Let *a*_1_ and *a*_2_ be two different activities defined by a set of sensors of size |*a*_1_| and |*a*_2_|, respectively. We define the EMD between *a*_1_ and *a*_2_ as follows:

(8) $$\text{EMD}(a_{1},a_{2})=\sum_{s_{1}\in a_{1},s_{2}\in a_{2}}\frac{\text{EMD}({\hat{P}}_{s_{1}},{\hat{P}}_{s_{2}})}{\left|a_{1}\right|\times \left|a_{2}\right|}$$

### 3.2. Semantic Similarity Graph (SSG)

**Figure 4.**
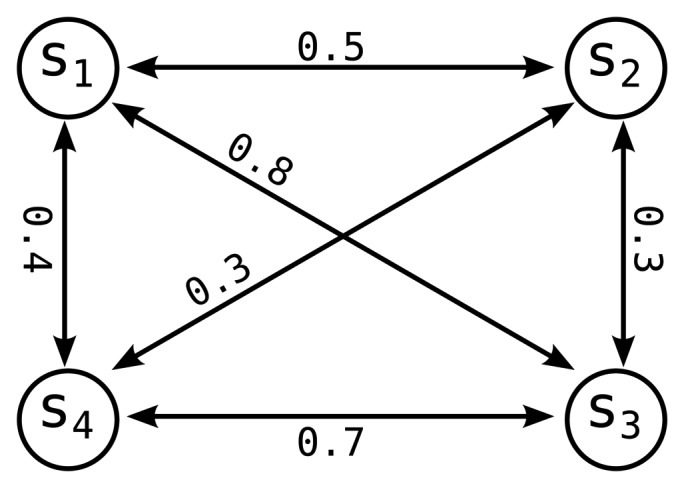
An example instance of the semantic similarity graph (SSG).

The EMD between a sensor pair is only computed when the probability functions of these sensors change significantly. Pairwise EMD results are stored in a complete graph called the semantic similarity graph (SSG). By means of SSG, the semantic similarity between two sensors can be fetched in constant time without performing any run-time computation. SSG stabilizes as the coverage of the accumulated long-term history increases. In order to allow system adaptation, we use the exponentially weighted moving average (EWMA) to accumulate the long-term behavior of sensors rather using a simple mean calculation. Figure 4 shows an example of an SSG instance containing four sensors, where each node represents a sensor and each edge between two sensors is the EMD between those sensors. SSG is updated if the behavior of a sensor changes significantly, for that specific sensor, and when the system is idle. According to our evaluations, our algorithm is not sensitive to inaccuracies in the SSG; thus, real-time updates are not necessary.

### 3.3. Cloaking Activities and Privacy

After the sensors in $a_{w_{k}}^{*}$ are determined based on predictions from the long-term history and the request messages received during the polling period, SSG is used to generate a cloak *AC_w_k__* to be used during *w_k_*. The level of protection due to *AC_w_k__* is measured in terms of the semantic difference between *AC_w_k__* and *AC_G_* = {*s*_1_, *s*_2_,…, *s_n_*}, *i.e.*, an activity cloak, including all sensors. We define *θ* = *EMD*(*AC_w_k__*, *AC_G_*) as a metric to measure the information leakage due to energy savings [5]. If the activity cloak is extended to include all sensors, *i.e.*, *AC_w_k__* = *AC_G_*, then *θ* = 0 and the privacy protection is maximum, because all of sensors are included in the cloak. Reducing the number of elements in *AC_w_k__* increases the *θ* value and reduces the energy consumption. For our experiments, we determine the *θ* value by using one-week training data to find the *θ* value that corresponds to the intersection of energy and privacy curves as explained in Section 4. We assume that both *AC_w_k__* and *AC_G_* refer to activities. Specifically, neither the fact that *AC_w_k__* contains sensors indicating one or more activities, nor the labels for them are relevant from the perspective of the algorithm. Based on the *θ* value and Equation (8), an activity cloak *AC_w_k__* with the maximum number of sensors is generated, such that:

(9) $$\sum_{s_{1}\in AC_{w_{k}},s_{2}\in AC_{G}}\frac{\text{E}({\hat{P}}_{1},{\hat{P}}_{2})}{\left|AC_{w_{k}}\right|\times \left|AC_{G}\right|}\leq \theta$$

The pseudo code for activity cloak generation is given in Algorithm 2. At the beginning of each time window, *w_k_*, CC adds sets of sensors defining one of the activities identified by the naive Bayesian classifier until the semantic distance between the *AC_w_k__* and *AC_G_* exceeds the predefined *θ* value.



**Algorithm 2:** The algorithm for generating activity cloaks.
 Let 
$AC_{w_{k}}=\{a_{w_{k}}^{*}\}$; Let *emd* = 0; **repeat**  Choose activity *a* with smallest *EMD*(*AC_W_k__*, *a*);  Add *a* to *AC_w_k__*;  Let *emd* = *EMD*(*AC_W_k__*, *AC_G_*); **until**
*emd* ≤ *θ*;


### 3.4. Lower Layer Considerations

The proposed algorithm does not make assumptions on the MAC or network layer protocols on the sensor nodes. This allows our scheme to work with different types of radios supported by various sensor boards. However, if the underlying MAC protocol supports TDMA, such as the case with the Bluetooth or ZigBee devices, then more energy can be saved by getting rid of the polling. On the other hand, since we reduce the number of active sensors substantially, the packet collusion probability is smaller relative to semantic blind traffic cloaking techniques [23].

## 4. Evaluation

### 4.1. Experimental Setting

**Figure 5.**
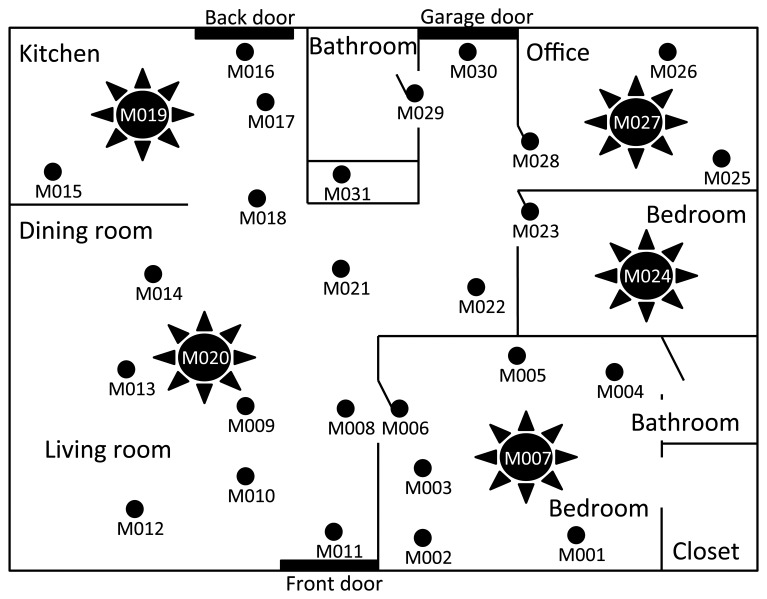
The floor and sensor deployment plan in a smart home environment.

We use a publicly available ADL monitoring data set [14]. The data set is annotated with activity labels and contains sensor readings and corresponding timestamps. The resident is an elderly woman who lives by herself, and her relatives visit the apartment regularly. The sensor data have been accumulated for about a year using 31 sensors deployed according to a topology in Figure 5.

To evaluate the performance of proposed privacy protection scheme, we compare our method against existing state-of-the-art privacy protection methods:

(1) Periodic transmission (*P*): all sensors transmit real or fake data according to a predefined period, at a constant rate. At each transmission period, the sensor sends real data if it senses an event since the last period.
(2) Exponential FPRtransmission (*E*): sensors follow an exponential distribution according to a predefined mean value. Real data packets are handled with shorter delay according to the algorithm given by Shao *et al.* [23].

We measure and compare the energy efficiency and privacy level of each protection scheme. We measure the transmission overhead of each approach by computing the ratio of the total number of fake data packets to the total number of real data packets and use this metric, called fake data ratio (FDR), as an indication of the energy overhead. Since the number of radio transmissions is inversely proportional to the network lifetime and the amount of real data transmissions are equal for all approaches, FDR is a fair and clear indication of the energy efficiency of the tested approaches. We use the FATS attack accuracy as a metric to evaluate the privacy benefits of tested approaches. For this purpose, we have implemented the FATS attack as described in Section 2. In these experiments, the adversary tries to identify each activity in real time and labels these activities using the FATS attack. The accuracy of the attack, then, is defined as the ratio of total number of actual activities observed during the experiment to the total number of activities correctly labeled by the attacker. Additionally, we also measure the maximum and average real data latency to show the overhead of privacy protection in the time domain.

### 4.2. Evaluation Results

We first evaluate the coverage of our activity cloaks. We use, as a metric, an unexpected event rate (UR), which is computed by dividing the number of unexpected events by the total number of events occurring during a day and, hence, is inversely proportional to the coverage; the higher the UR, the lower the coverage, and *vice versa*. For statistically meaningful results, we randomly select one hundred dates from the data set and apply the proposed method by varying the size of *w_k_* under 3 different choices for the training period. Furthermore, ACs are generated using *θ* = 1.0, which restricts each AC to only carry the predicted ADLs and sensed activity requests that are made in the previous time window. By excluding the cloaking activities, we can directly evaluate the performance of ADL prediction.

**Figure 6.**
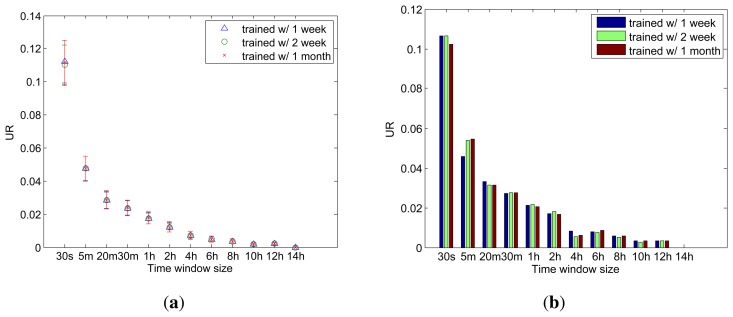
Activity cloak coverage: the unexpected event rate (UR) *vs.* the time window size. (**a**) 100 random days; (**b**) Day 169.

As shown in Figure 6a, where each data point represents an average UR from 100 random days, while each bar indicates a standard deviation, UR decreases as *w_k_* increases. While a larger *w_k_* is preferred for better coverage, one should consider the fact that the maximum latency suffered by an unexpected event is equal to the size of *w_k_*. On the other hand, the training period has little impact on the performance when the behavioral pattern of a resident is fairly regular. Please note that the best training period would depend on the lifestyle of a resident. For comparative evaluations, we also select and evaluate a specific day, 169th; as shown in Figure 6b, a specific behavioral pattern from a certain day does not deviate much from the general patterns that the resident has. These evaluation results demonstrate that *w_k_* should be at least 5 min to achieve the ADL prediction accuracy of 95% or higher and that it can meet this accuracy requirement after one week of training. Note that under the actual scenario, *θ* will be lower than 1, causing more sensors to be included in AC, thus leading to almost zero UR.

A smaller *w_k_* causes higher overhead, since the computational complexity of EMD is *O*(*N*^3^*logN*), where *N* equals the number of time windows throughout a day [24]; e.g., using a 10-min window requires approximately 330-times higher computational costs than a one-hour window. However, this computational cost is acceptable when EMD and AC computations are done off-line at the central server. To further optimize the computational cost, a kernel distance metric [25] may be used as an alternative to EMD.

**Figure 7.**
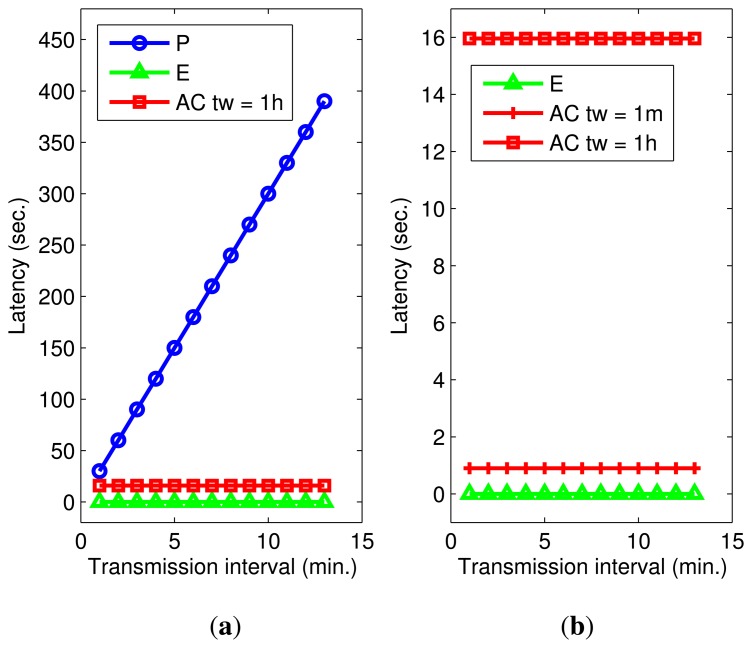
Latency of privacy protection methods.

Unlike the exponential-based approach, the proposed method has non-zero probability of having unexpected events, which may incur additional delay. To evaluate this, we observe the latency of all three methods for the 169*th* day. For this experiment, *θ* is fixed at 1.0, while a transmission interval varies. The transmission interval represents a period for *P* or a mean value for the probabilistic distribution used in *E* and AC. Figure 7a plots the result of latency evaluation, which demonstrates that periodic transmission has the highest latency proportional to the period reaching up to 6.5 min. On the other hand, the latency of transmitting according to an exponential distribution is constant and slightly over one second. The AC method behaves like *E* in that the latency is constant regardless of the mean interval, and the observed maximum latency is 16 s with one-hour time windows. Figure 7b compares AC for 1-min and 1-h time window sizes with *E* by varying transmission intervals. According to the experimental results, the average delay per packet is 1 s when the window size is 1 min. We conclude that the proposed privacy protection method using the AC mechanism delivers an order of magnitude smaller latency than the periodic-based method while suffering from slightly longer delays than that of the exponential-based one. The latency is closely related to the coverage of the activity cloak, and it continuously improves after system start-up.

**Figure 8.**
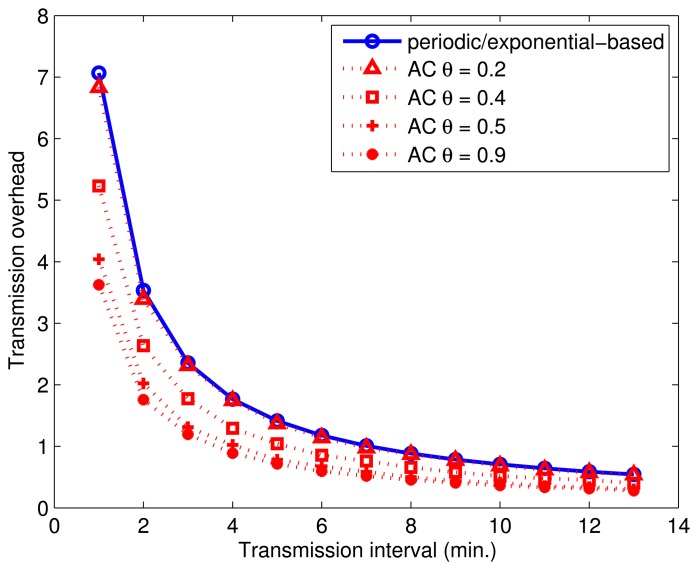
Energy overhead of the tested approaches.

We next compare the energy efficiency of baselines *P* and *E*, against AC by varying transmission intervals. Two baselines have very similar energy characteristics when the test duration is sufficiently longer than the transmission interval. This is expected because *E* uniformly distributes inter-transmission delay over the interval, and the average number of packets is equal to the amount of packets sent when transmissions are made at equidistant intervals, which is the behavior of *P*. Figure 8 shows the average change in FDR when the transmission interval is varied from 1 min to 14 min for *P*, *E* and AC. We plot the performance of AC with various values of *θ* to show the effects of this variable on the energy overhead. According to Figure 8, the FDR value decreases logarithmically in all tested approaches when the transmission interval is increased. We observe that the energy overhead of AC (*θ* = 0.2) is very similar to *P* and *E*. For such a low value of *θ*, all of the sensors are included in the cloak, so essentially, the AC algorithm becomes virtually the same as the *E* algorithm. Increasing the *θ* to 0.5 reduces the energy overhead by 45% regardless of the transmission interval, due to the reduced number of cloaking activities in the AC. The energy overheads of *P* and *E* are more than twice compared to that of AC (*θ* = 0.9). These observations indicate that by controlling the *θ*, we can control the energy overhead of our algorithm.

**Figure 9.**
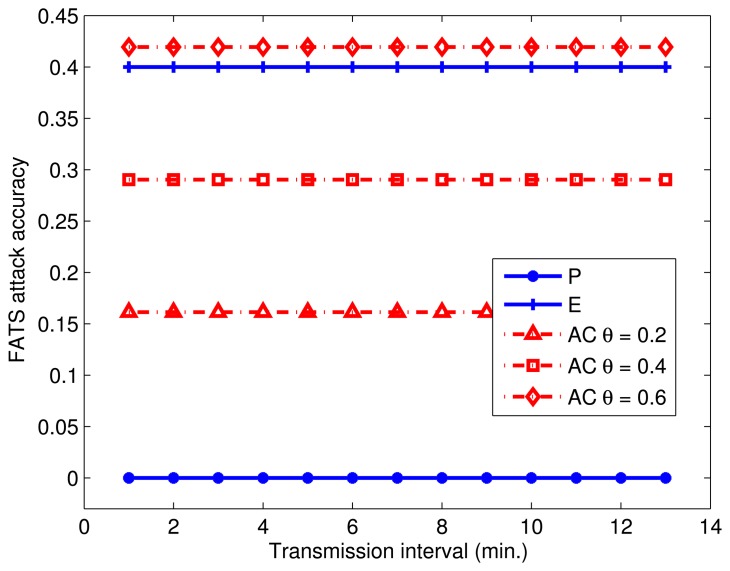
Privacy level of different protection methods.

We then compare the privacy level provided by the tested approaches. For this experiment, we again vary the transmission interval from 1 min to 14 min and compare the Tier 3 accuracy of *P*, *E* and AC (*θ* = 0.2, *θ* = 0.4, *θ* = 0.6). Figure 9 summarizes the results of this experiment, where the y-axis denotes the average ratio of successful ADL identifications to the total attempts of the adversary. We observe that *P* provides the highest level of protection under FATS attack with a prediction accuracy close to 0 regardless of the transmission interval. This is because every possible sensor uniformly transmits data, and the FATS attack can neither cluster nor label any sensors. The Tier 3 accuracy of the FATS attack under *E* is 40%, which is slightly better than the performance of AC (*θ* = 0.6), yielding a FATS accuracy of 42%. When the protection method is AC, the prediction accuracy of the FATS attack is 16% and 29% for *θ* values of 0.2 and 0.4, respectively. Although transmitting according to an exponential distribution significantly reduces the real-data latency, the prioritization of these packets also reveals more information on the ADLs, due to behavioral changes in the sensors detecting events. This observation confirms the findings of Shao *et al.* [23]. The accuracy of the FATS attack under AC is proportional to the value of *θ*, while the performance of AC counter intuitively lies somewhere in between *P* and *E*. Before the experiments, we expected AC to perform at most as good as *E*, since the underlying transmission mechanism is identical; however, FATS attack accuracy is significantly higher with *E* than it is with AC. The reason for this behavior is that the FATS attack fails to do accurate classification when some of the sensors, *i.e.*, those that are excluded from the cloak, disrupt the exponential distribution, which significantly hinders clustering accuracy.

**Figure 10.**
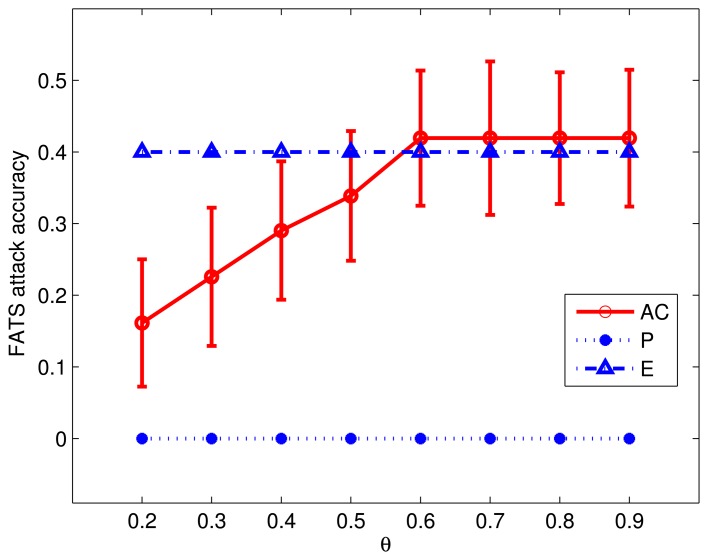
Activity cloak (AC) method privacy level dynamics.

We further investigate the relationship between *θ* and FATS attack Tier 3 accuracy in a separate experiment. In this experiment, we vary the *θ* in the range [0.2, 0.9] and measure the Tier 3 accuracy as the ratio of successful activity predictions over total predictions. For each value of *θ*, we randomly select 100 days from the data set and report the mean accuracy of these runs. Experiment results, as summarized in Figure 10, confirm that the information leaked to the intelligent adversaries is inversely proportional to the changes in *θ*. Moreover, the accuracy of the attack saturates at 44% for *θ* ≥ 0.6. Such saturation depends on the number of sensors deployed in an environment. The granularity and range of *θ* will increase as the number and variety of sensors in the environment increases. In Figure 10, the relative performance of AC when compared to that of *P* and *E* indicates that AC performs almost as good as *E* independent of the value of *θ*, while *P* always provides better protection.

**Figure 11.**
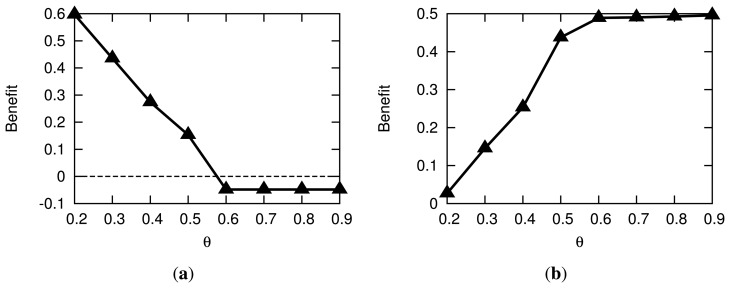
Privacy and energy benefits of AC over Exponential FPR transmission (*E*). (**a**) Privacy benefit; (**b**) Energy benefit.

In Figure 11, we summarize the privacy and energy gains due to the AC method when the baseline is *E*. The maximum privacy benefit is achieved for smaller values of *θ*. Specifically, when *θ* = 0.2, the relative privacy benefit compared to *E* is 60% (Figure 11a). When *θ* = 0.3, the benefit reduces to 40%, and this trend continues until *θ* = 0.57, at which point the AC method starts to perform slightly worse than *E*, until it eventually saturates at *θ* = 0.6. Figure 11b shows the energy benefit of AC compared to *E*. In this case, AC performs better than *E* for all tested values of *θ* with a maximum of approximately 50% less energy dissipation when *θ* ≥ 0.6. As we decrease the value of *θ*, the proposed method increases the number of sensors in the activity cloak; therefore, the energy benefit decreases. The minimum relative gain of 0.3% over *E* is observed when *θ* = 0.2. Since both privacy and energy gains saturate for *θ* ≥ 0.6, we conclude that the feasible range for *θ* is [0.2,0.6]. However, it is worth repeating that this range depends on the number and variety of sensors in the environment.

### 4.3. Discussions

According to the experimental results, *θ* can control the energy efficiency and the privacy protection level of our algorithm. Although periodic transmission seems to provide the best protection, it is worth noting that, given the limited number of possible activities in a smart home environment, the accuracy of ADL prediction can never be zero, and the results we provide depend heavily on the mechanisms of the FATS attack. Furthermore, a prediction accuracy of 50% is too low to be actually useful.

Our contribution focuses on generating privacy-aware activity cloaks to maximize the energy efficiency, and hence, our proposed method is orthogonal to an underlying mechanism of packet scheduling. In our implementation, we prefer the exponential FPR scheduling to the periodic scheduling to minimize the per-packet latency. It is noted that the computational cost of exponential FPR scheduling is higher than that of periodic scheduling. While the periodic mechanism simply delays the real data transmissions according to a predefined period, the exponential FPR uses a procedure to determine a proper value of delay, not violating the predetermined probabilistic distribution, which requires *O*(*nlogn*) computations, where *n* is the number of previous transmissions.

The activity cloaking method can still conceal the actual activities under the assumption that the adversary has accurate information on the habitual patterns of the inhabitants along with the sensor locations by means of, e.g., social engineering or some advanced filtering mechanism. This is because cloaking activities are selected based on their semantic similarities to the actual activity. For example, during our experiments, we observe that the system starts pairing shower and bedroom activities together in the early hours of the day. This is due to the small EMD value between these activities. Since EMD is a reflexive relation, the existence of one of these activities in the activity cloak implies the existence of the other activity. Therefore, the adversary will not be able to identify the actual activity between equally-likely activities in the cloak. We observe the same relationship, for example, between watching TV and dining, sleeping and bathroom activities. Furthermore, most of the time, there are multiple activities in the cloak paired together, and these pairings adapt to changes in the daily lives of the residents, due to the updates to the SSG.

Internet-enabled devices and appliances, such as TV sets and fridges, may also expose certain activity information. Although we specifically focus on wireless sensor nodes in this work, the proposed scheme can be implemented on such devices with minimal cost. Furthermore, these devices are generally not battery-powered, so traditional traffic obfuscation techniques can also be applied to them.

## 5. Related Work

The privacy and security of wireless sensor networks (WSNs) have been studied extensively [26–29]. Specifically, data eavesdropping [30], source anonymity [28] and false data injection [7] attacks have been addressed. Increased research efforts on smart environments brought about different concerns for privacy, such that these concerns have been identified as an obstacle against widespread adoption of smart home systems [3]. The privacy protection method described in this paper protects against a side channel attack that depends on time signatures and radio fingerprints rather than the encrypted data [31] to infer daily activities, such as cooking or showering [4]. The attack first determines individual sensors by identifying distinct fingerprints of radio transmitters. Once unique data sources are identified, they are grouped together into clusters based on the spatial proximity of data transmissions, where each cluster represents a physical room or a logical association. For example, most of the sensors located in various parts of the dining room detect movement within a small time window, and sensors in the kitchen are also activated during dinner. Labels for these clusters, such as “dining room” or “kitchen”, can be inferred by using inter-cluster and intra-cluster activation patterns. The cluster labels and traffic patterns reveal sufficient information on the sensors, such that individual identification becomes possible. With this information, the attacker can label each sensor with a location and a type, such as a contact sensor in the bathroom. Activities can be then predicted with high accuracy based on the cluster and sensor labels, along with the transmission patterns. The whole scheme is based on radio fingerprinting, which is the identification of the data source based on imperfections in the signal due to physical differences in different transmitters [4,13,15,17]. Protection against fingerprinting requires resource-hungry, complex hardware [4]. Despite the necessity of fingerprinting for activity identification, it is not a threat against privacy by itself.

The focus of this work is on hiding the patterns that are used in clustering and type identification of wireless sensor nodes. Previous work on hiding transmission patterns mostly uses fake messages transmitted in between data messages. In [8], a method based on periodic transmissions has been described. According to [8], each sensor is assigned a time slot at which it must transmit a fake or data message depending on the availability of data. However, it has been shown that this method introduces significant delays [23]. To reduce large delays introduced by periodic transmission, a probabilistic method called Fit Probe Rate (FPR) has been proposed [23]. In FPR, each data source transmits data or fake messages at intervals that fit into an exponential distribution, but a minimum possible delay is assigned to data messages. This method guarantees an order of magnitude lower delay for data messages compared to the periodic scheme when the mean delay in the FPR is equal to the message interval in the periodic scheme. Both periodic and probabilistic transmissions against traffic analysis attacks require continuous fake data injections by all sensors, hence having considerable energy overhead [32]. An alternative method based on k-anonymity [33] was proposed in [34]. According to the proposed method in [34], semantically meaningful noise is injected into the wireless network to create fake events over the actual transmission data, such that real sensor data is sent in real time, without any delays and the adversary observes multiple events. One drawback of this approach is the complexity of generating fake events that are indistinguishable from actual events. In our scheme, we instead use FPR slots to overcome this problem. Although there is a clear trade-off between privacy protection, network lifetime (energy efficiency) and response time (latency) [35], previous work in the literature fails to provide a means to control this trade-off and bias towards one aspect, such as reducing latency, to improve the protection overhead. The approach we propose in this work exposes a parameter called the *θ* value, by means of which the level of privacy can be controlled.

The conventional method for protecting against traffic spoofing is injecting fake data or introducing random delays in order to obscure the transmission patterns. Since latency has a significant impact on the user experience, fake data generation is preferable in a smart home environment. In order to limit the energy consumption of our scheme, we devise an algorithm that is similar to the cloaking areas used in location-based services (LBS). Cloaking areas are used in the domain of mobile computing in order to provide privacy, but still allow individuals to benefit from an LBS [5,36,37]. According to this, an approximate cloaking area is reported to service providers instead of the exact location of the user. The construction of a cloaking area is based on k-anonymity or l-diversity. In k-anonymity-based methods, multiple users' behavior is projected to the service provider, whereas schemes based on l-diversity increase the semantic scope of the location information.

High volumes of long-term smart home data are also processed for extracting patterns and information, especially in the domain of healthcare systems [38–40]. The proposed method is completely orthogonal to the work in the big-data domain, because the fake transmissions are known and filtered out by the system. As a result, the sensor data is never tainted, and any analysis on the historical or real-time data will produce results that it would generate in a privacy blind setting. Mission critical services, such as healthcare systems and harm prediction engines, require real-time processing and actuation [40–42]. While periodic transmissions introduce large delays to sensor reports and, hence, are unfeasible for mission critical deployments, the FPR packet scheduler we use for traffic obfuscation guarantees the delivery of real sensor data within one tenth of the mean transmission delay [43]. Furthermore, events that are extremely critical can be reported immediately, since in those cases, privacy becomes a secondary concern, and they are rare enough to be ignored in the evaluation of any algorithm.

## 6. Conclusions and Future Work

This paper introduces a light-weight, energy-efficient, low-latency privacy protection scheme for smart home environments against side channel attacks. The proposed method expands the semantic scope of the information an adversary can gather by sniffing the encrypted wireless communication. We introduce the concept of cloaking activities in order to reduce the energy overhead of existing protection algorithms. The experiments using real data collected from a smart home environment indicate up to a 50% reduction in the energy dissipation and orders of magnitude decreases in the per-packet latency compared to previous methods. We also show that the proposed approach can substantially reduce the accuracy of the FATS attack. The proposed method exposes the trade-off between privacy and energy efficiency via the system parameter *θ*. In the future, we plan to extend the algorithm to adapt *θ* based on the system conditions without user interference by classifying activities in privacy groups, as well as to support environments with multiple residents by expanding the activity prediction set.
